# Direct imaging of Indium-rich triangular nanoprisms self-organized formed at the edges of InGaN/GaN core-shell nanorods

**DOI:** 10.1038/s41598-018-34382-y

**Published:** 2018-10-30

**Authors:** Gordon Schmidt, Marcus Müller, Peter Veit, Sebastian Metzner, Frank Bertram, Jana Hartmann, Hao Zhou, Hergo-Heinrich Wehmann, Andreas Waag, Jürgen Christen

**Affiliations:** 1 Institute of Physics, Otto-von-Guericke-University Magdeburg, Magdeburg, Germany; 2 Institute of Semiconductor Technology and Laboratory for Emerging Nanometrology LENA, Technische Universität Braunschweig, Braunschweig, Germany

## Abstract

Higher indium incorporation in self-organized triangular nanoprisms at the edges of InGaN/GaN core-shell nanorods is directly evidenced by spectral cathodoluminescence microscopy in a scanning transmission electron microscope. The nanoprisms are terminated by three 46 nm wide a-plane nanofacets with sharp interfaces forming a well-defined equilateral triangular base in the basal plane. Redshifted InGaN luminescence and brighter Z-contrast are resolved for these structures compared to the InGaN layers on the nanorod sidewalls, which is attributed to at least 4 % higher indium content. Detailed analysis of the *inner* optical and structural properties reveals luminescence contributions from 417 nm up to 500 nm peak wavelength proving the increasing indium concentration inside the nanoprism towards the nanorod surface.

## Introduction

Semiconductor nanorods provide a promising platform for multi-functional applications in optoelectronics as efficient light emitting diodes (LED)^1–7^, nanolasers^8^, functionalized sensors^9–11^ and positioned single photon sources^12–14^. In particular, coaxial InGaN/GaN nanorods benefit from their unique three-dimensional geometry with high aspect-ratios, offering a substantially increased optical active area, the growth of defect free material, as well as non-polar surface orientations. Additionally, dislocation bending inside the nanostructures leads to an extremely reduced dislocation density, resulting in an exceptional material quality of the nanorods^15^. Despite the many benefits of nanorod heterostructures, the growth of three-dimensional core-shell nanostructures is more complex in comparison to that of planar two-dimensional films. A detailed understanding of the growth process and the indium incorporation in the non-polar shell layers is crucial to achieve uniform compositions for highly efficient devices.

Combining low temperature cathodoluminescence (CL) spectroscopy with scanning transmission electron microscope (STEM) offers the unique possibility to correlate local optical and compositional structural properties with nanometer spatial resolution^16–18^. Thus, a direct nanoscopic insight into the growth mechanism of ternary InGaN layers around three-dimensional nanorods is possible and presented in this work.

## Methods and Sample Setup

**Figure 1:**
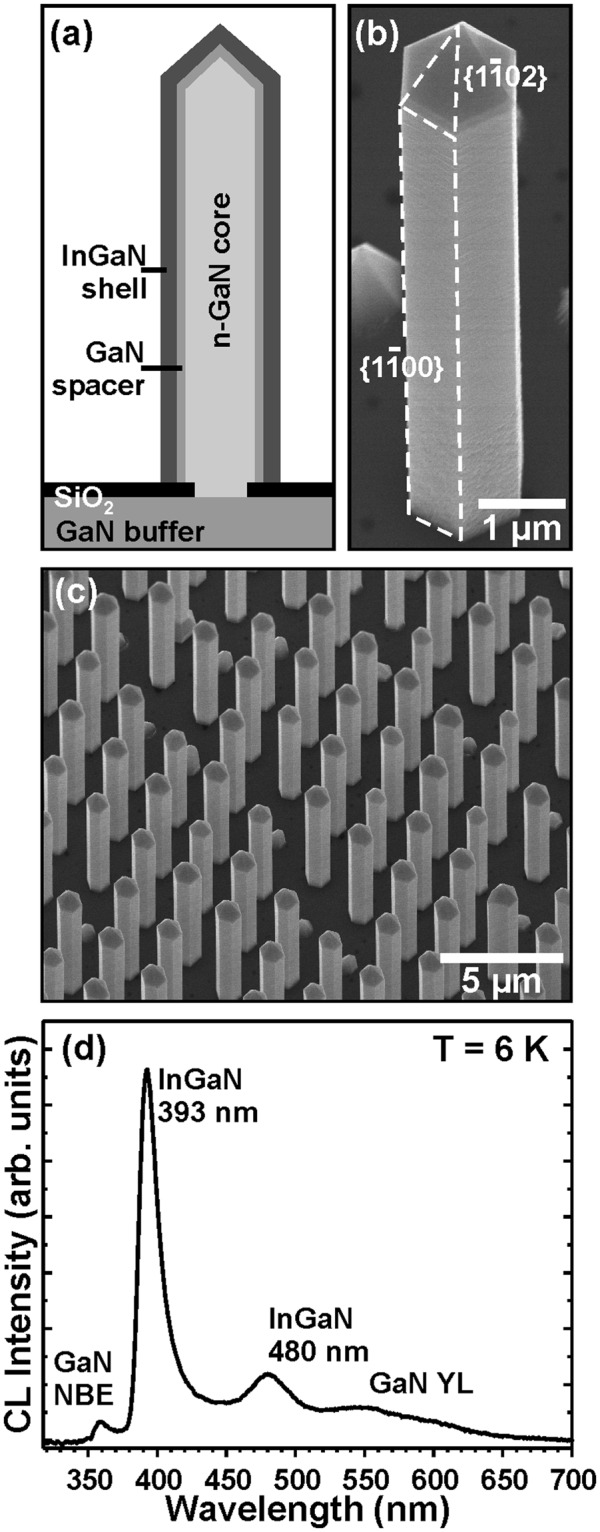
(**a**) Cross-section scheme of the nanorod heterostructures, (**b**) SEM image of a single nanorod with labeling its sidewall facets, and (**c**) the as-grown nanorod array in bird’s eye view; (**d**) spatially averaged CL spectrum of nanorod ensemble measured at T = 6 K.

The three-dimensional InGaN/GaN core-shell nanorod array under investigation has been produced by continuous-mode metal-organic vapor phase epitaxy (MOVPE) using selective area growth (SAG) approach. 2-inch patterned SiO_x_ on GaN/sapphire substrates have been used as SAG templates. The 100 nm thick SiO_x_ masking layer was fabricated by plasma-enhanced chemical vapor deposition (PECVD). Using photolithography and reactive ion etching circular holes were opened into the SiO_x_ in a hexagonal lattice with an aperture diameter and pitch distance of 0.8 µm and 2.4 µm, respectively. Subsequently, three-dimensional Si-doped GaN nanorods were firstly grown at 1040 °C under pure H_2_ carrier gas using trimethylgallium (TMGa) and ammonia (NH_3_) as precursors. To achieve a high aspect ratio the growth of the nanorods is performed in a two-step process: first, the vertical growth rate was increased by using a high silane (SiH_4_) flow rate of 110 nmol/min and a low V/III ratio of 77, which differs from planar GaN growth conditions^19,20^. In a second step, the SiH_4_ flow rate was decreased to reduce SiN_x_ passivation of the n-GaN core surface. Next, an intrinsic GaN spacer was deposited around the Si-doped GaN core. Finally, an InGaN shell layer with a nominal thickness of 30 nm was grown enfolding the GaN core structure. The growth temperature for InGaN shell layer fabrication were set to 750 °C under N_2_ atmosphere and using a very high V/III ratio of around 12,000. A schematic drawing of the core-shell nanorod heterostructure is depicted in Fig. 1(a) together with an SEM image of an individual column in Fig. 1(b).

Using a liquid helium sample holder, low temperature CL spectroscopy (T < 20 K) was performed in a scanning transmission electron microscope FEI (S)TEM Tecnai F20 to analyze the local optical and structural properties. In STEM mode the electron beam is focused onto the TEM lamella at an acceleration voltage of 80 kV. The generated cathodoluminescence is coupled out of the microscope by a parabolically shaped mirror into the grating monochromator MonoCL4 (Gatan) and detected by a nitrogen cooled Si-CCD. Detailed information about our STEM-CL setup can be found elsewhere^21–23^. The STEM specimens were prepared by conventional mechanical polishing followed by Ar+ ion milling in a liquid nitrogen cooled precision ion polishing system. Supporting SEM-CL measurements were performed in our SEM-CL set-up described elsewhere^24^.

## Results and Discussion

Figure 1(c) shows a scanning electron micrograph (SEM) in bird’s eye view of an array of InGaN/GaN core-sell nanorods. The SEM measurements reveal a homogeneous selective area growth of three-dimensional nanostructures with high aspect ratios over the whole GaN/sapphire template. The nanorods are exclusively grown out of the mask openings, no residual deposition of material on top of the SiO_x_ layer is observed, indicating a high selectivity of the growth process. Rare local deviations, i.e. formation of pyramids instead of nanorods, are probably due to inhomogeneity in the gas phase, shading effects and/or passivation of the pyramids during initial growth stage. The nanorods cover 25 % of the planar surface with a mean density of 2 × 10^7^ rods/cm^2^. The average diameter and height of the nanorods was measured to be 970 nm and 7.3 µm, respectively, resulting in a high aspect ratio of 7.5. A close-up view of a single nanorod can be seen in Fig. 1(b), where the well-defined hexagonal shape of the structure is clearly visible. Six non-polar {1–100} sidewalls and a pyramidal tip with semi-polar {1–102} facets terminate the nanorod.

The spatially averaged SEM-CL spectrum of the nanorod ensemble in Fig. 1(d) at T = 6 K is dominated by InGaN emission with two broad peaks at 393 nm and 480 nm. Due to the excitation of the underlying GaN spacer and the GaN core, the near band edge (NBE) as well as the yellow luminescence is obtained as well.

**Figure 2:**
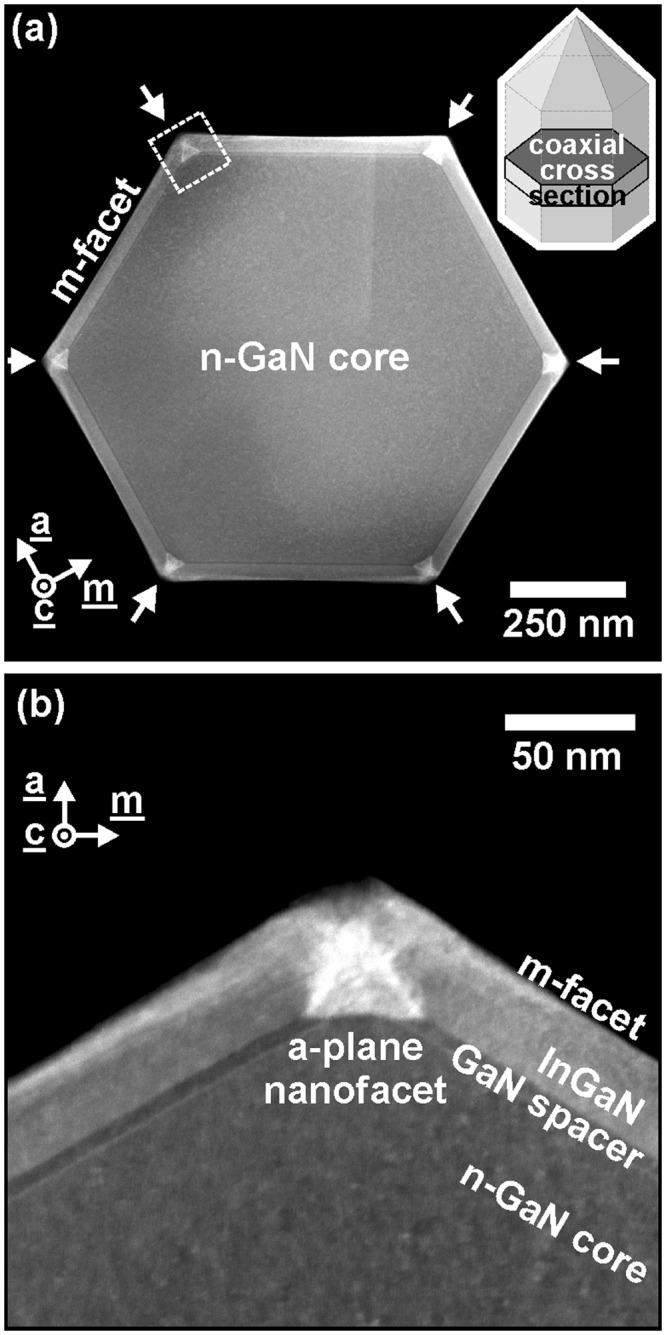
(**a**) HAADF image of the coaxial cross-section (inset: sketch of nanorod section) of a single nanorod in overview as well as (**b**) in higher magnification. The area of the magnified image (**b**) is marked in the overview (**a**) in the upper left nanorod corner by a white dashed line. The complete layer structure is resolved: the n-GaN-core, the following GaN spacer and the thick InGaN layer. A brighter HAADF contrast is found at the edges of the nanorod hexagon (marked by arrows in (**a**)) evidencing the formation of In-rich triangular nanoprisms.

The nanoscopic real structure of the InGaN/GaN core-shell nanorods was examined by analytical scanning transmission electron microscopy (STEM). Figure 2(a) shows the STEM micrograph in high-angle annular dark-field (HAADF) contrast of a single nanorod in coaxial cross-section geometry perpendicular to the [0001]-direction. As the HAADF contrast is primarily given by the atomic number of the analyzed material, dark regions in the image correspond to GaN and brighter regions to InGaN with different [In] (brighter contrast corresponds to higher [In]). The complete radial shell structure is clearly identified in the STEM image. Beginning with the nanorod center, the whole Si-doped GaN core (inner and outer core) appears homogeneously with a diameter of (895 ± 5) nm from {1–100} facet to {−1100} facet. STEM analysis verified the absence of extended defects like threading dislocations or stacking faults in the core. Enfolding this n-GaN core a thin shell layer appears in brighter contrast, indicating the possible formation of a passivation layer during initial core growth^25^. As previously reported by Hartmann *et al*., the three-dimensional growth of nanorods under high silane flow can promote the formation of such SiN_x_ passivation layer^19^. Finally, the GaN spacer decorates the m-plane core facets and the InGaN shell layer enfolds the complete GaN nanorod with sharp interfaces. The thickness of the GaN spacer and InGaN shell layer on the {1–100} facet are (5.4 ± 0.5) nm and (32.5 ± 0.5) nm, respectively. Structural measurements yield an identical layer thickness on all non-polar facets, proving uniform growth rates and a consistent selective area epitaxy.

Most strikingly, during the InGaN growth, nanoprisms with triangular cross-section are formed at the six vertices of the hexagonal cross-section (see white arrows in Fig. 2(a)). The brighter contrast of the triangles in the HAADF image evidences a higher indium content in comparison to the m-plane shell layer. Figure 2(b) shows a HAADF-STEM image of one of the hexagonal edges at higher magnification (position marked in Fig. 2(a)). At the edges of the GaN core a {11–20} a-plane with a length of (46.3 ± 0.5) nm has been formed. The formation of a-plane nanofacets at the edges has been previously reported for self-assembled GaN nanowires grown by molecular beam epitaxy^26,27^. Three crystallographic equivalent {11–20} facets forming an equilateral triangle with 60° internal angles, terminating the In-rich nanoprism.

**Figure 3:**
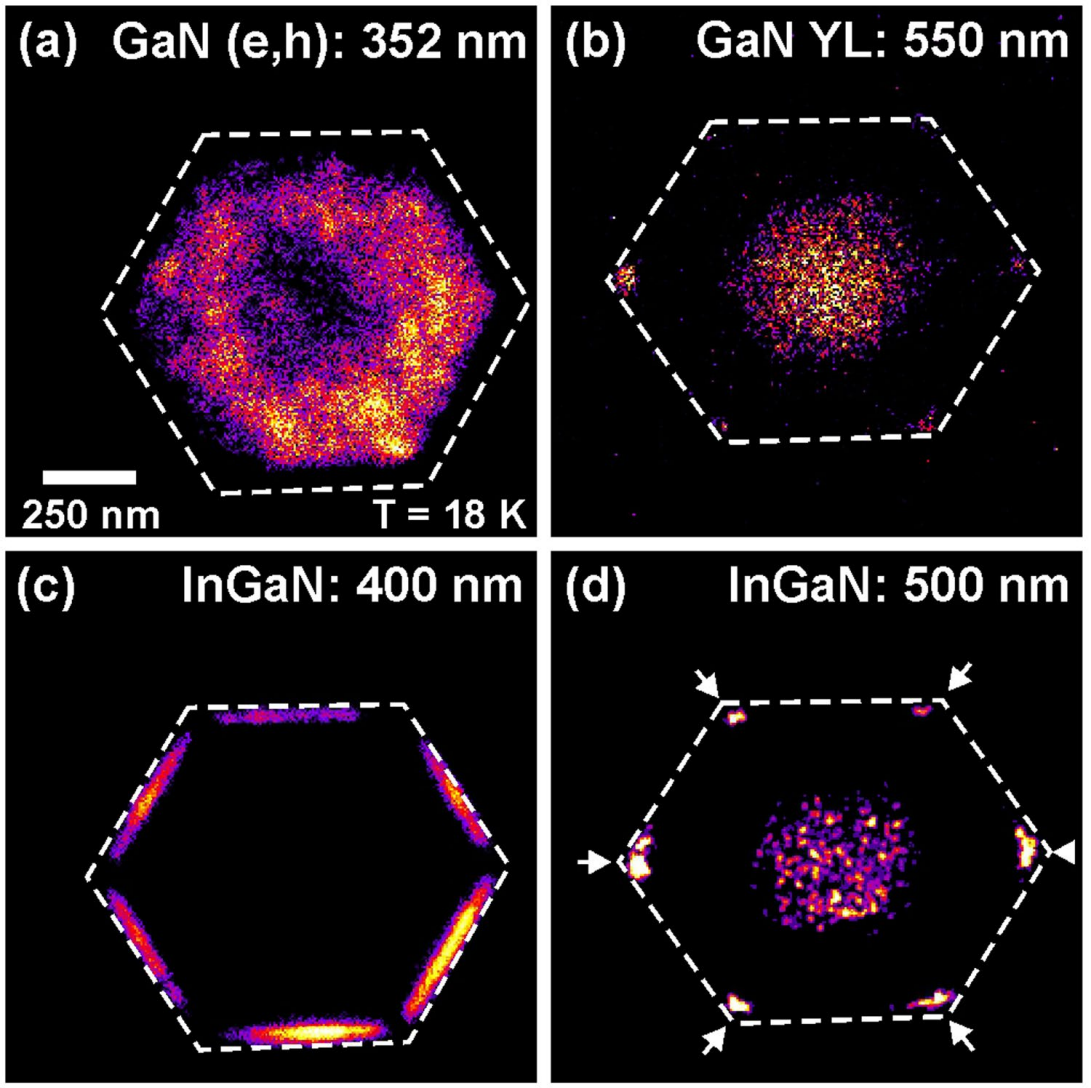
Monochromatic CL intensity distribution at 18 K for (**a**) GaN (e, h) recombination (λ = 352 nm), (**b**) GaN yellow luminescence (λ = 550 nm), (**c**) m-plane InGaN (λ = 400 nm), and (**d**) InGaN nanoprism (λ = 500 nm), respectively.

The luminescence properties of the nanorod are directly imaged by nanometer-scale cathodoluminescence mapping. Figure 3(a–d) shows monochromatic CL maps of the dominant recombination channels at low temperature (T = 18 K) recorded together with the corresponding HAADF image in Fig. 2(a). Figure 3(a) and (b) concentrates on the GaN emission, visualizing the strong impact of the high Si doping of the inner core. Yellow luminescence (YL) only appears in the center part of the GaN core but not outside (Fig. 3(b)), whereas intense band-band transition luminescence at 352 nm occurs in the outer region only (Fig. 3(a)). The band-band transitions are attributed to recombinations of free electrons in the conduction band with holes at the valence band maximum^28–30^. Due to a high free carrier concentration in the highly silicon doped core leading to band filling and exciton screening, the (e, h) recombination dominates the near band edge emission. In contrast, the YL is connected to point defects accompanied with deep levels incorporated during the growth with high silane flow rate, which are apparently denser in the inner core and reduce in density towards the outer region of the doped GaN core. The reason for this difference is that the central part of the core is grown in c-direction whereas the outer part grows in semi-polar direction. This results in different densities of point defects which is clearly visualized with the monochromatic intensity distributions of the (e, h) recombination and yellow defect luminescence.

The lower row of Fig. 3 depicts two monochromatic CL images of the InGaN spectral region taken at 400 nm (Fig. 3(c)) and at 500 mn (Fig. 3(d)). All six m-facets yield intense CL emission at the main InGaN peak (λ = 400 nm) over the whole thickness of the InGaN shell except the very corners. Exactly at these corners of the nanorod and exclusively there, the intensity of the 400 nm InGaN emission drops down and longer wavelength InGaN CL (λ = 500 nm) appears. The formation of such In-rich InGaN nanoprisms and their redshifted luminescence in respect to the m-plane InGaN has been reported before by Griffiths *et al*.^27^.

**Figure 4:**
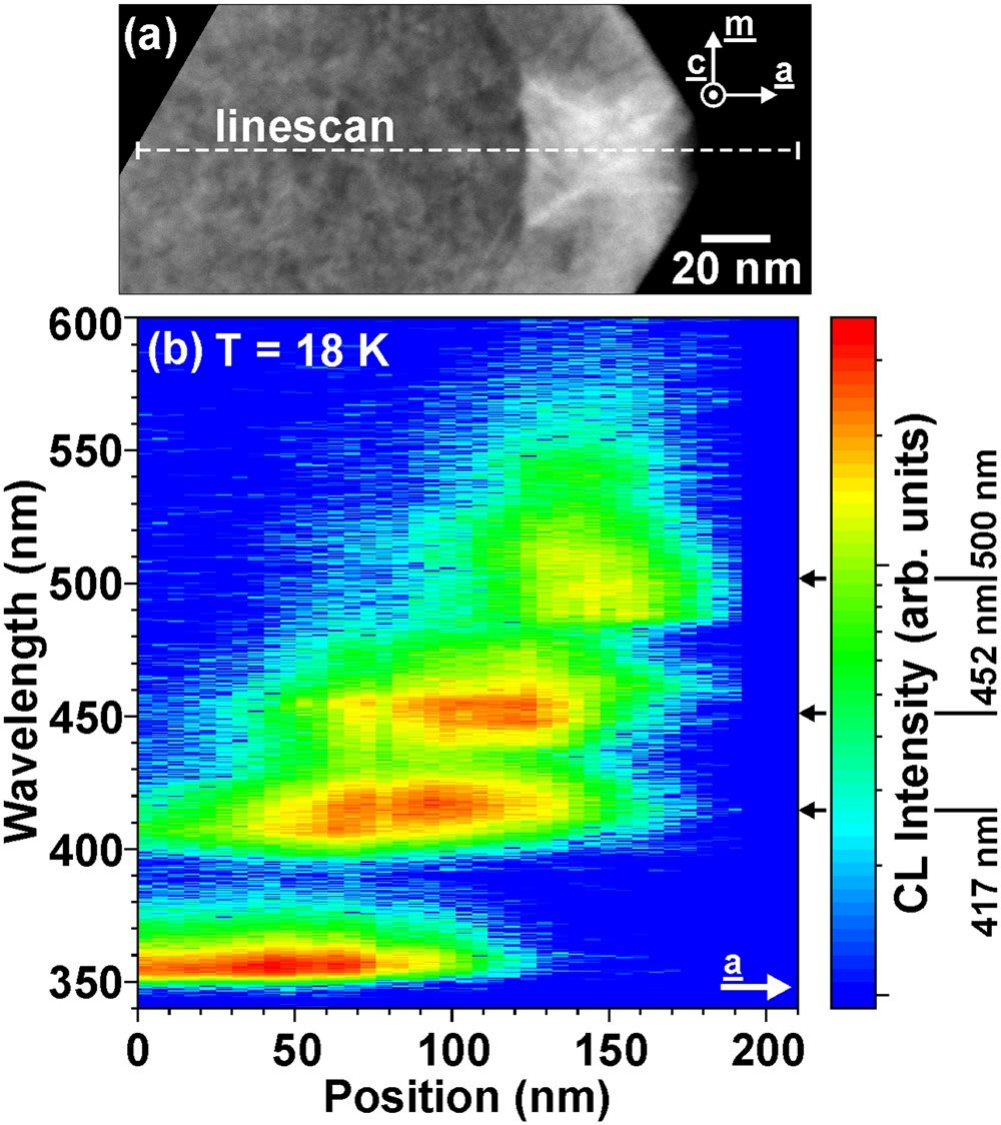
(**a**) HAADF image of a single nanoprism with (**b**) STEM-CL spectrum linescan along a-direction from the GaN core across the nanoprism (see dashed line in (**a**)), which exhibits clearly three different InGaN emission channels at 417 nm at the bottom, 452 nm in the middle part and 500 nm at the top.

For a further insight into the indium incorporation within these nanoprisms, a spectrum linescan in a-direction was performed across an individual nanoprism (see Fig. 4). Starting in the GaN core a dominant GaN (e, h) emission can be observed (λ = 355 nm). Towards the surface three spatially and spectrally well separated InGaN luminescence contributions can be resolved. Luminescence at 417 nm is emitted close to the interface between the GaN core and the a-plane InGaN layer (see Fig. 4(b)). This peak wavelength of 417 nm corresponds to a calculated indium concentration of ~17 % (for a bulk InGaN layer pseudomorphically strained on a-plane GaN (transition energies of pseudomorphically strained a-plane InGaN alloys were taken from^27^ by changing a- and m-direction resulting in equivalent band energies from Pikus-Bir kp-theory)). Subsequently, a redshifted InGaN CL at 452 nm appears, followed by a weak cathodoluminescence at λ ~ 500 nm close to the surface. The reduced intensity of the 500 nm emission can be attributed to the enhanced nonradiative recombination of generated carriers at the nearby surface as well as lower crystal quality (see Fig. 4(a)). Different In incorporation can also be concluded from the HAADF image (see Fig. 4(a)), where we see a brightening of Z-contrast towards the surface. This is qualitatively in agreement with the redshifted InGaN emission in a-direction. The quantitative strain and In distribution as well as possible quantization effects are not clear for this complex structure.

**Figure 5:**
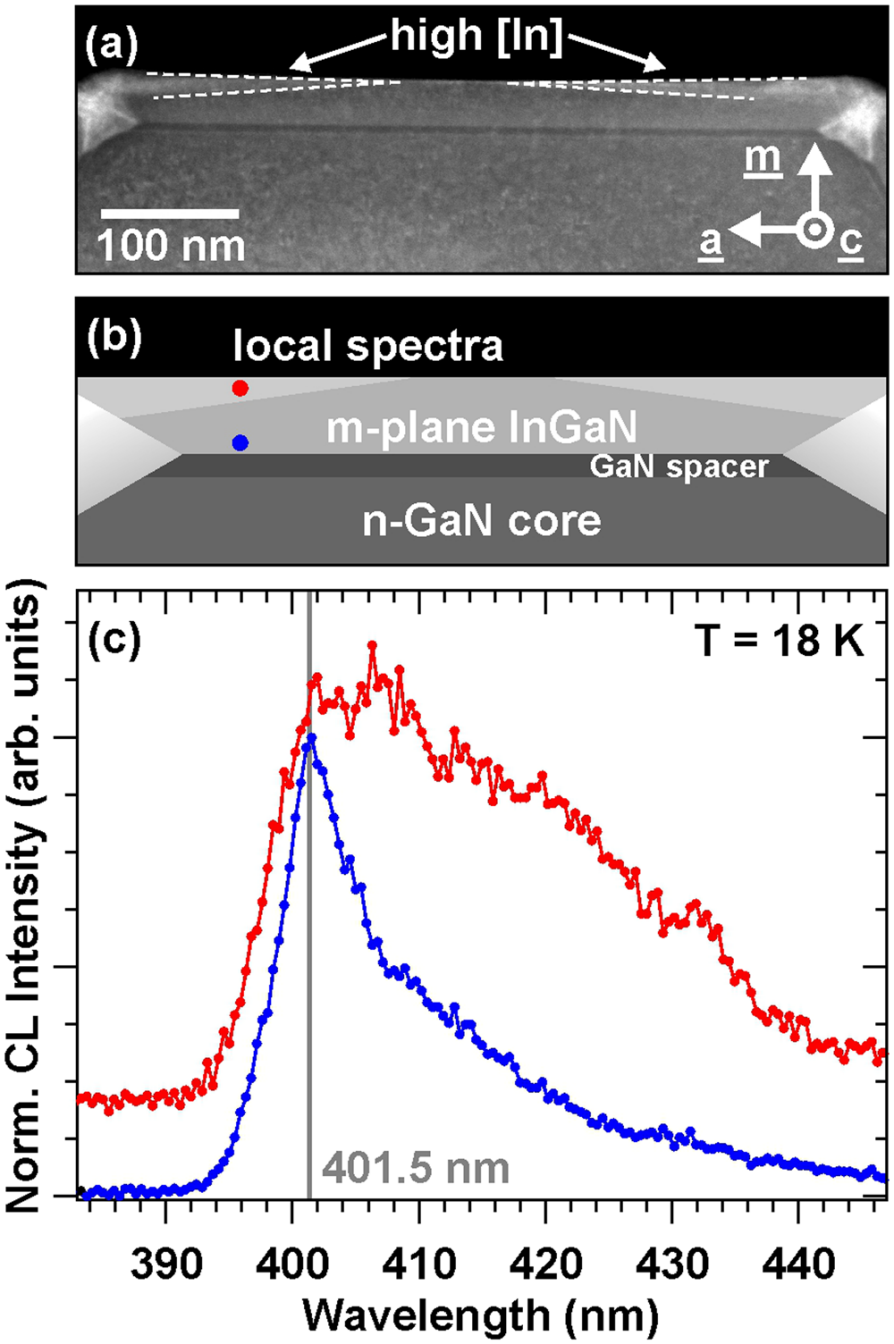
(**a**) HAADF coaxial cross-section image of m-plane InGaN with brighter Z-contrast at the wings towards edges (marked by arrow/dashed white line), (**b**) schematic drawing of region with different In concentrations and position of local spectra, (**c**) local spectra from m-plane InGaN close to the interface (blue) and high [In] region at the wings (red).

For comparison, an HAADF image of the m-plane InGaN shell layer is shown in Fig. 5(a). In respect to the nanoprisms, we observe darker Z-contrast for InGaN grown on GaN spacer indicating a lower In content (see Fig. 5(a)). A local CL spectrum directly acquired at the InGaN/GaN spacer interface exhibits an emission at 401.5 nm (see Fig. 5(c)). Compared to the nanoprism CL, this drastically blue shifted emission clarifies the lower indium content in m-plane InGaN shell layer. The peak wavelength of 401.5 nm gives an estimation of [In] ~ 13 % for the anisotropically strained m-plane InGaN pseudomorphically grown on GaN^31^. So approximately 4 % less indium is incorporated into the first layers of m-plane InGaN-shell with respect to the InGaN nanoprisms.

Concentrating on the m-plane InGaN close to the surface, we observe wedges of brighter contrast in HAADF images with clearly defined boundaries to the underlying InGaN material (see markings in Fig. 4(a)). The wedge thickness increases from the facet-center towards the nanorod edge. The Z-contrast enhancement is related to higher indium concentration compared to InGaN material growing directly on top of the GaN spacer, which is verified by local CL spectra (see Fig. 5(b)). In contrast to the first InGaN layers grown on m-plane GaN spacer (blue spectrum in Fig. 5(b)), we observe an emerging low energy shoulder in the wedge domain shifting the overall emission peak to 406.3 nm verifying a higher indium concentration.

As evidenced by HAADF and CL, the InGaN shell forms complex domains of different In content around the GaN nanorod. Even within these domains different indium concentrations are observed, as shown in the nanoprisms. Issues like the different indium incorporation on different crystal facets^32–39^, the sophisticated strain distribution for three dimensional grown structures^40–45^ as well as surface conditions^25^ and atomic configurations^39,46^ could have a significant influence on the indium incorporation in InGaN nanorod shell layers. We want to point out that the growth mechanism of these domains is much more complex compared to the proposed model of Griffiths *et al*.^27^. They attribute higher indium incorporation solely to elastic strain variations. This, however, contradicts with the formation of well defined, sharp interfaces between the different InGaN regions. Our HAADF-CL findings clearly indicate that the current explanations for the creation of the In-rich regions are incomplete. Due to the three dimensional structure of the nanorod, more degrees of freedom are present during the epitaxial deposition of the InGaN shell. In particular at the vertices, the layer-by-layer growth is interrupted and a three dimensional growth at the 16 hexagon edges might result in the selforganized triangular nanoprisms.

In summary, we investigated the indium incorporation in InGaN core-shell nanorods by low temperature cathodoluminescence performed in scanning transmission electron microscopy. Self-organized Indium-rich triangular nanoprisms are formed at the edges of InGaN/GaN core-shell nanorods terminated by a-plane nanofacets and exhibit strongly redshifted luminescence in respect to the m-plane side walls. Inside the nanoprisms, three spatially separated InGaN emission contributions from 417 nm up to 500 nm peak wavelength towards the nanorod surface indicate the lattice-pulling effect. At least 4 % higher indium concentration is concluded for the triangular shaped region compared to the m-plane sidewalls. Additionally, close to the surface of the m-plane InGaN domains of higher indium incorporation are resolved. This study clearly illustrates a self-organized complex indium accumulation for thick InGaN shell layers grown on GaN nanorod core.

## Data Availability

The datasets generated and analysed during the current study are available from the corresponding author on reasonable request.
